# Gene expression suggests conserved aspects of *Hox *gene regulation in arthropods and provides additional support for monophyletic Myriapoda

**DOI:** 10.1186/2041-9139-1-4

**Published:** 2010-07-05

**Authors:** Ralf Janssen, Graham E Budd

**Affiliations:** 1 Department of Earth Sciences, Palaeobiology, Villavägen 16, SE-75236 Uppsala, Sweden

## Abstract

Antisense transcripts of *Ultrabithorax *(a*Ubx*) in the millipede *Glomeris *and the centipede *Lithobius *are expressed in patterns complementary to that of the *Ubx *sense transcripts. A similar complementary expression pattern has been described for non-coding RNAs (ncRNAs) of the *bithoraxoid *(*bxd*) locus in *Drosophila*, in which the transcription of *bxd *ncRNAs represses *Ubx *via transcriptional interference. We discuss our findings in the context of possibly conserved mechanisms of *Ubx *regulation in myriapods and the fly.

Bicistronic transcription of *Ubx *and *Antennapedia *(*Antp*) has been reported previously for a myriapod and a number of crustaceans. In this paper, we show that *Ubx/Antp *bicistronic transcripts also occur in *Glomeris *and an onychophoran, suggesting further conserved mechanisms of Hox gene regulation in arthropods.

Myriapod monophyly is supported by the expression of *aUbx *in all investigated myriapods, whereas in other arthropod classes, including the Onychophora, *aUbx *is not expressed. Of the two splice variants of *Ubx/Antp *only one could be isolated from myriapods, representing a possible further synapomorphy of the Myriapoda.

## Background

The Hox genes are expressed in broad overlapping domains along the anterior-posterior axis of developing arthropods, and specify the segment identity under the control of upstream acting segmentation genes [1,2]. In *Drosophila*, the initially established expression patterns of the Hox genes are maintained by the trithorax (trxG) and Polycomb group (PcG) factors [3]. These factors act through sets of response or maintenance elements (MEs), the best investigated of which are involved in the regulation of the *Ultrabithorax *(*Ubx*) gene [4,5]. A number of non-coding RNAs (ncRNAs) have been reported for *Drosophila*, which are transcribed through MEs in the *bithoraxoid *(*bxd*) region located between *Ubx *and *abd-A*. The ncRNAs including *bxd *are expressed in similar patterns to those of the neighbouring Hox genes [6,7]. Although it was initially thought that *bxd *would activate *Ubx*, a recent study suggests that transcription of ncRNAs promoted by Trithorax represses *Ubx *in *cis *by means of transcriptional interference [4]. Elongated transcription of *bxd*-ncRNAs through the *Ubx *locus prevents the transcription of the latter in the same cells. However, in cells that do not express *bxd Ubx *is expressed [4]. The expression patterns of *bxd *ncRNAs and *Ubx *are therefore complementary in *Drosophila*.

In organisms other than *Drosophila*, the mechanisms that regulate *Ubx *transcription are less well known. It is unclear whether MEs or *bxd *are conserved or if transcription of *bxd *interferes with the transcription of *Ubx *in a similar way to that in *Drosophila*. However, some evidence has recently accumulated suggesting that a similar mechanism could be involved in the regulation of *Ubx *outside *Drosophila*. Data from the beetle *Tribolium *show that ncRNAs of the *Ubx *region are expressed in patterns similar to those of the neighbouring Hox genes, resembling the observations in *Drosophila *[8]. In the centipede *Strigamia*, the non-coding antisense transcript of *Ubx *is expressed in a pattern complementary to that of the coding *Ubx *sense transcript, suggesting that bidirectional transcription of a non-coding RNA, antisense *Ubx*, is also involved in the regulation of *Ubx *in this myriapod [9].

In this paper, we present data from two distant myriapod relatives - the millipede *Glomeris marginata *and the centipede *Lithobius forficatus *- which show conserved expression of antisense *Ubx *(*aUbx*) in a pattern complementary to that of *Ubx *in Myriapoda. Data from species of other arthropod groups and the onychophoran *Euperipatoides kanangrensis *reveal that *aUbx *expression does not represent an ancestral feature but a synapomorphy of the Myriapoda. The latter provides support for the still controversially discussed idea that the Myriapoda form a monophyletic group [10].

An mRNA that encodes a single protein, which describes the typical case for eukaryotic genes, is termed monocistronic, whereas mRNAs encoding two or several proteins are termed bicistronic and polycistronic respectively. We show here that bicistronic transcripts of *Ubx *and *Antp *(*Ubx/Antp*), as described for a number of crustaceans and the centipede *Strigamia *[9,11], also exist in *Glomeris *and *Euperipatoides*. This finding suggests that bicistronic transcription is an ancestral feature that is likely to be involved also in arthropod Hox gene regulation by means of transcriptional interference and the blockade of *Antp *translation.

## Materials and methods

### Species husbandry and embryo treatment

The general handling of *G. marginata *is described in Janssen *et al. *[12]. The embryos were allowed to develop at room temperature (22 to 25°C). The developmental stage of the embryos was determined by 4'-6-diamidino-2-phenylindole (DAP) staining. Staging was performed as described previously [12,13].

Specimens of *L. forficatus *were collected from a leaf litter stack in the backyard of the Evolutionary Biology Centre (EBC) in Uppsala/Sweden in spring (May/June). Around 50 centipedes were held at room temperature in a spacious plastic box filled with washed leaf litter (washing away small particles makes the later finding of the eggs easier). The adults were fed with pieces of common earthworms (*Lumbricus*) every few days. The often detritus-covered eggs were collected by hand and incubated in plastic dishes on damp paper tissues until they reached the desired developmental stage. Staging was performed as described previously [14]. Generally, the handling was carried out similarly to the method described for *Lithobius atkinsoni *[15].

Embryos of the spider *Cupiennius salei*, the onychophoran *E. kanangrensis *and the red flour beetle *Tribolium castaneum *were obtained and treated as described previously ([16-18], respectively).

### Gene cloning

Fragments of *Ubx *and *Antp *transcripts of *G. marginata *were obtained via 5' and 3' rapid amplification of cDNA ends (RACE)-PCR (Gene Racer RACE Kit; Invitrogen, Carlsbad, CA, USA). A fragment (383 bp) of *Tribolium Ubx *corresponding to the C-terminal end of the open reading frame (ORF) (94 bp) and the beginning of the 3' untranslated region (UTR) was isolated with gene-specific primers (Table 1). General Hox primers, as described previously [19], were used to isolate a small fragment of *Ubx *from *Euperipatoides *cDNA. An extended fragment was subsequently obtained by 3'-RACE.

**Table 1:** Primers used for PCR.

Gene	Direction	Primer sequence 5' → 3'
*Tribolium Ubx*	Forward	CCCAATTACGTATATAGTTG

	Reverse	GATCAAAGAACTCAACGAGC

*Lithobius forficatus Ubx*	Forward	GGAGGAGGCGGATAGAGATG

	Reverse	TTAATTGGTTTGGGTAGGGG

*Artemia Ubx/Antp*	Forward (1)	TACCTGACGAGACGAAGG

	Reverse (1)	CTCTTTCTTCCATTTCATTCG

	Forward (2)	CAGATCAAGATATGGTTCC

	Reverse (2)	GTCAAACATAAAGCATGGG

*Euperipatoides Ubx/Antp*	Forward	GCCGAAGGATAGAAATGGCTCACGC

	Reverse	CCGAGTGTACGTCTGCCTTCCTCG

*Glomeris Ubx/Antp*	Forward	GCGGAGGAGGCGGATAGAAATGG

	Reverse	TTTTAATCTGGCGTTCCGTCAGGC

*Tribolium Ubx/Antp*	Forward (1)	GGAAAAAGAGTTCCACACAAA

	Reverse (1)	CCCCATTTCGCATGTCCG

	Forward (2)	GATCAAAGAACTCAACGAGC

	Reverse (2)	GATCTGTCTTTCGGTTAAAC

	Forward (3)	CAGGCTCAAAAAGCGGCG

	Reverse (3)	Against N-terminal part of ANTP

A fragment of *Lithobius forficatus Ubx *was isolated with gene-specific primers based on the published sequence of *Lithobius atkinsoni Ubx *[15]. The isolated *L. forficatus *fragment is only 221 bp long, but works well in hybridization experiments.

Part of the bicistronic transcripts containing *Ultrabithorax *and *Antennapedia *(*Ubx/Antp*) were isolated from the brine shrimp *Artemia *(first PCR), the onychophoran *Euperipatoides *and the millipede *Glomeris*. The gene-specific primers used were directed against the homeodomains of *Ubx *(forward primer) and *Antp *(backward primer). Gene-specific primers to amplify a possible *Tribolium Ubx/Antp *transcript failed, even though we used the primers (Table 1) in all possible combinations including nested PCRs.

Sequences of the fragments were determined from both strands by sequencing (Big Dye Terminator Cycle Sequencing Kit; Perkin-Elmer Applied Biosystems, Foster City, CA, USA) chemistry on an automatic analyser (ABI3730XL; Perkin-Elmer Applied Biosystems) by a commercial sequencing service (Macrogen, Seoul, Korea). Sequences are available in GenBank under the accession numbers FN687748 (*Gm-Ubx*), FN687749 (*Gm-Antp*), FN687750 (*Gm-Ubx/Antp*_variant II), FN687751 (Ek-Ubx), FN687752 (*Ek-Ubx/Antp*_variant I), FN687753 (*Ek-Ubx/Antp*_variant II), FN687754 (*Lf-Ubx*) and FN687755 (*Af-Ubx/Antp*_variant II).

### *In situ *hybridization and nuclear staining

Whole-mount *in situ *hybridization for all species was performed as described previously for *Glomeris *[20]. Double whole-mount *in situ *hybridization and cell nuclei detection using DAPI was performed as described by Janssen *et al. *[21]. Embryos were analyzed under a dissection microscope (Leica, Heerbrugg, Switzerland) equipped with a digital camera (Axiocam; Zeiss, Jena, Germany) or a DC100 (Leica) digital camera. Brightness, contrast and colour values were corrected in all images using image processing software (Adobe Photoshop CS2., V.0.1 for Apple Macintosh; Adobe Systems Inc. San Jose, CA, USA).

## Results

### *Ultrabithorax *and *Antennapedia *transcripts

Partial sequences of the transcripts of all ten Hox genes of *G. marginata *were published previously [19]. In all cases except *fushi-tarazu*, only part of the homeodomain and 3' UTR sequence was obtained. The published *Ubx *fragment neither ends in a poly-A tail nor has one of the typical polyadenylation sites and is therefore likely to be incomplete. Recent 3'-RACE experiments demonstrated the presence of additional 3' UTR transcript. The extended fragment ends in a poly-A tail, but lacks an obvious polyadenylation site close to this. The 3' UTR region contains nine possible polyadenylation sites more distant from the poly-A tail, allowing for the presence of transcripts with different 3' UTR length. Whether the recovered '3' UTR' sequence is a typical UTR that occurs in the monocistronic transcript of *Ubx *or if is merely the result of the bicistronic transcript of *Ubx *and *Antp *(see following section) is unclear.

**Figure 1:**
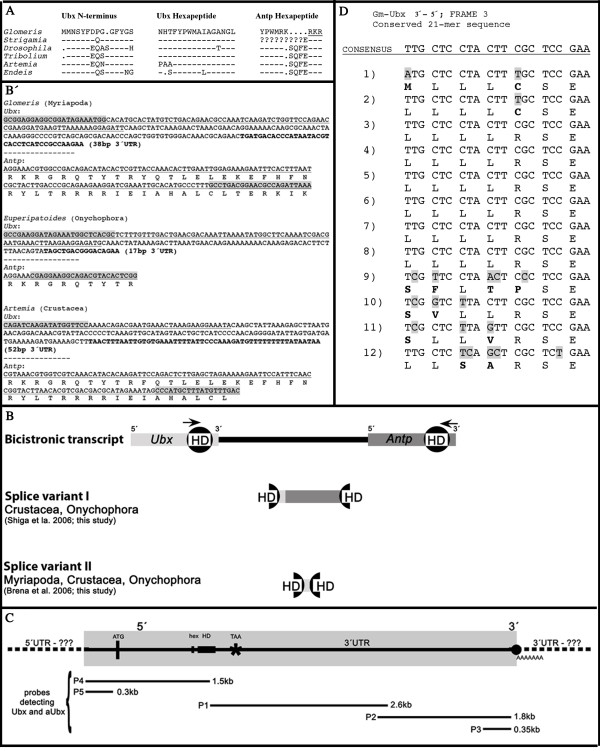
**Sequence information on Ubx, Antp and Ubx/Antp**. (A) Conserved N-terminal region of UBX and hexapeptide sequence of UBX and ANTP in arthropods. Dashes indicate conserved positions, dots represent gaps, question marks stand for unknown sequence. Amino acids contributing to the homeodomain are underlined. (**B) **Overview of bicistronic transcripts of *Ubx/Antp *and their splice variants in arthropods. *Ubx *sequence is in light grey; *Antp *sequence is in dark grey; positions of primers for the detection of *Ubx/Antp *are indicated by arrows. In splice variant I, *Antp *is almost exactly abutting the open reading frame of *Ubx *with only few base pairs of *Ubx *3' UTR in between. In splice variant II, all sequence of *Antp *5' to the homeodomain (HD) is missing. **(B) **Sequences of the *Ubx/Antp *splice variant II from *Glomeris*, *Euperipatoides *and *Artemia*. The homeodomain is underlined, primers are shaded, and the 3' UTR of *Ubx *is in bold. **(C) **Extension of isolated *Ubx *mRNA and inferred extension of the *aUbx *transcript. Probes (P1 to P5) detecting *Ubx *and *aUbx *are indicated (not to scale). Whether 5'- and 3' UTR transcripts extend beyond the detected area is unclear (question marks). Positions of start codon (ATG), hexapeptide (hex), homeodomain (HD), stop codon (TAA) and poly-A tail (dot__AAAAAA_) are indicated. **(D) **Twelve conserved 21bp-repeats situated in the 3' UTR of *Glomeris Ubx*. The sequences are abutting each other without bases in between. Consensus sequence is on top. Differences from the consensus are marked by shaded bases, changed amino acids are in bold.

We recovered 5'-RACE fragments of *Ubx *and *Antp*. The *Ubx *fragment represents the complete N-terminal region of the ORF and 5' UTR sequence. The 5'-*Antp *fragment is incomplete and does not include the N-terminal region of the protein coding sequence and the 5' UTR. The fragments encode conserved motifs that are characteristic for *Ubx *and *Antp *orthologs in arthropods (Figure 1A). Note that the *Glomeris *ANTP protein lacks the characteristic SQFE motif between the hexapeptide and the homeodomain. Instead, this short peptide is replaced by a single lysine (K) in *Glomeris *(Figure 1A). The expression pattern of all newly recovered fragments is identical to those described previously [19] (not shown).

### Bicistronic transcript of *Ultrabithorax *and *Antennapedia*

For *Glomeris*, we identified an *Ubx/Antp *bicistronic transcript that encodes the *Ubx *homeodomain C-terminal to the upstream primer position and 38 bp of the *Ubx *3' UTR, which is directly adjacent to the complete N-terminal part of the *Antp *homeodomain up to the downstream primer position (splice variant II; see below) (Figure 1B,B'). Whether the sequence C-terminal to this sequence is part of the fusion transcript is unclear; however, the sequence N-terminal to the described short fusion transcript has been independently recovered by 5' RACE using gene specific primers (GSPs) against the *Antp *homeodomain that amplified the *Ubx/Antp *fusion transcript instead of the *Antp *5' transcript. This sequence is part of the *Ubx *transcript as proven by 5'-RACE PCR for *Ubx*.

We also successfully isolated a splice version (splice variant I) of *Ubx/Antp *bicistronic transcripts from an onychophoran (*Euperipatoides*). This splice variant I is also described for a number of several crustaceans including the brine shrimp *Artemia *[11] (Figure 1B). For *Euperipatoides *and *Artemia*, we also isolated the shorter splice variant II of the bicistronic transcript described for *Strigamia *[9] (Figure 1B,B'). A splice variant I is not described for *Strigamia *and we could not isolate it from *Glomeris *either. We failed to detect any *Ubx/Antp *bicistronic transcripts in the beetle *Tribolium *(Insecta).

### Extension and nature of the *Ubx *antisense (*aUbx*) transcript

The information on *aUbx *transcription is based on probes detecting the *Ubx *antisense strand during *in situ *hybridization experiments (Figure 1C). It was thus necessary to unravel the true extension of the *aUbx *transcript by *in situ *hybridization experiments with minimum size probes (around 300 bp for *Glomeris*) detecting *aUbx *complementary to the ends of the available *Ubx *fragments (Figure 1C). In all cases these sense probes detected the *aUbx *expression pattern (described below) suggesting their complete transcription. Whether the *aUbx *transcript extends the *Ubx *transcript is unclear; however, it does not extend into the transcripts of *abdominal-A *(*abd-A*) or *Antennapedia *(*Antp*), because *in situ *hybridization experiments with anti-*abd-A *and anti-*Antp *probes did not detect any transcription. The longest possible ORF of the *aUbx *transcript is 113aa long and encodes a repetitive sequence of the type (LLLLR/cSE) (Figure 1D).

### Expression of *aUbx*

**Figure 2:**
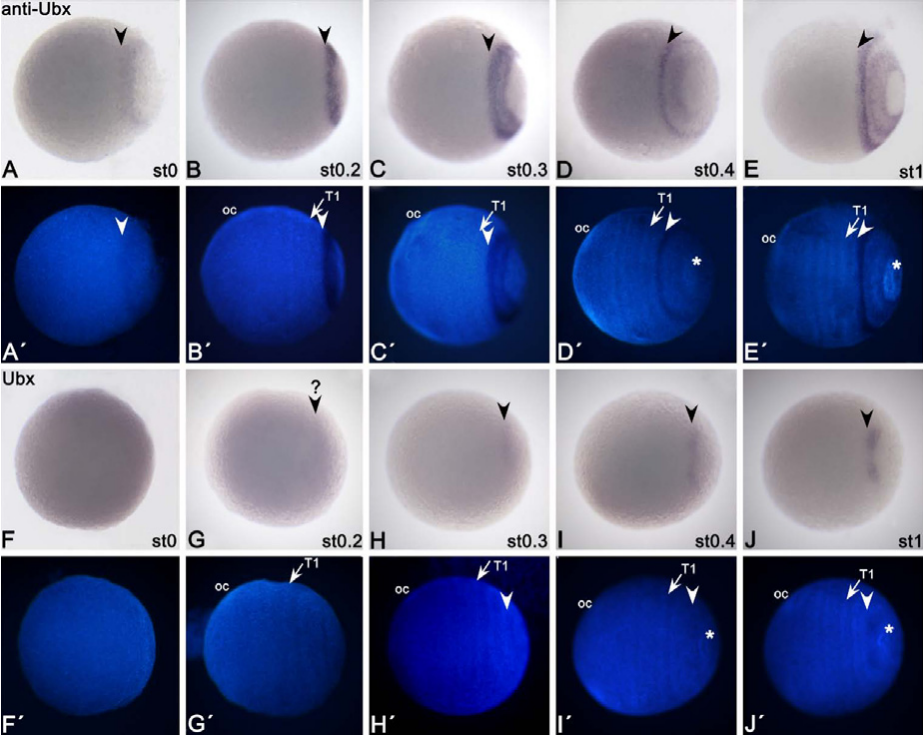
**Early expression of a*Ubx *and *Ubx *in *Glomeris marginata***. **(A-E) **Embryos expressing *aUbx*. **(A'-E') **DAPI counterstaining of the bright field images shown in (A-E). **(F-J) **Embryos of same stage as in (A-E) expressing *Ubx*. Arrowheads point in all cases to the border of anterior expression. Note that expression in (G) is almost below the detectable level (?). Arrows point to T1 segment. Asterisks demarcate tissue posterior to the growth zone that gives rise to the proctodaeum and hindgut. All embryos are shown with the anterior to the left. oc, Ocular segment (segment anterior to antennal segment); st, embryonic stage; T1, first trunk segment.

**Figure 3:**
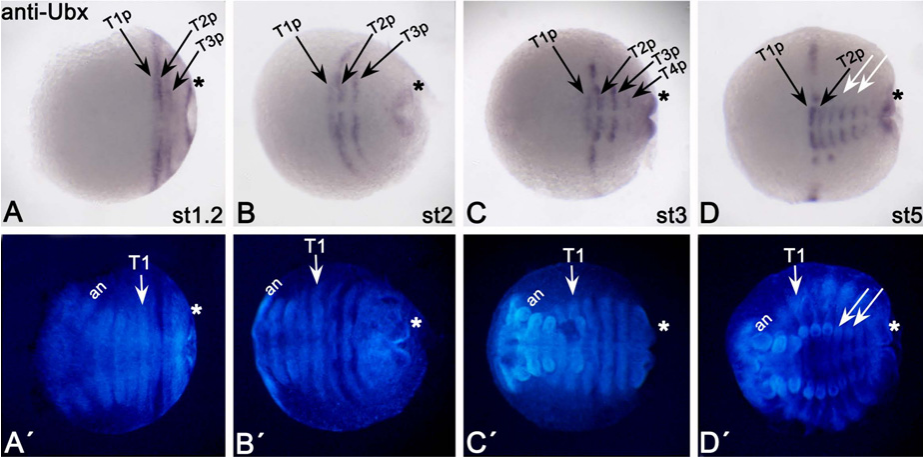
**(A-D) Late expression of *aUbx *in *Glomeris marginata***. All embryos have anterior to the left. Asterisks mark anal valves. White arrows point to lack of expression in limb buds of T4 and T5. an, Antennal segment; st, embryonic stage; T1-T3, first to third trunk segment; T2a, anterior part of T2; T2p, posterior part of T2.

Transcripts of *aUbx *can already be detected at the blastoderm stage in a broad posterior domain (Figure 2A); at stage 0.2, this expression intensifies (Figure 2B). At the next stage (0.3) the centre of the initial broad domain is cleared from the transcripts (Figure 2C). At stage 0.4, the domain splits into an anterior stripe and a broad posterior domain (Figure 2D). The broad domain lies anterior to the future proctodaeum; the anterior stripe covers the intersegmental indentation between trunk segment two (T2) and T3, and is thus located in the posterior part of T2. At stage 1, the posterior domain has broadened and its anterior and posterior margins show enhanced expression (Figure 2E). At stage 1.2, the complete T2 segment expresses *aUbx*, although the expression in its anterior part is weak (Figure 3A). The anterior margin of the former broad domain (Figure 2E) has now transformed into an independent stripe in the posterior of T3 (Figure 3A). The posterior-most expression is in the anal valves (AV). Ventrally, the expression of *aUbx *is weaker than in its corresponding lateral and dorsal tissue (Figure 3A). At the subsequent stage (stage 2) three stripes of *aUbx *expression are detectable: in the posterior areas of T1, T2 andT3 (Figure 3B). This expression is restricted to the ventral tissue only for the stripes in T1 and the T3, whereas the stripe in T2 extends into the dorsal tissue. All stripes are discontinuous at the ventral midline. At around stage 3, an additional stripe forms in the posterior of T4 (Figure 3C). In subsequent stages (4 to 6), additional discontinuous stripes of *aUbx *appear in the ventral germ band with the formation of additional segments. Expression in dorsal tissue, legs and anal valves remains unchanged. Expression of the anterior-most *aUbx *stripe (the posterior stripe in T1 (T1p)), is enhanced at these stages (Figure 3D and data not shown). Note that although the legs posterior to T3 are forming, *aUbx *is not expressed in their tips (Figure 3D). The posterior-most part of the developing early embryo, which will later give rise to the hindgut and the proctodaeum, remains free from *aUbx *expression (Figure 2).

### Complementary expression patterns of *Ubx *and *aUbx*

**Figure 4:**
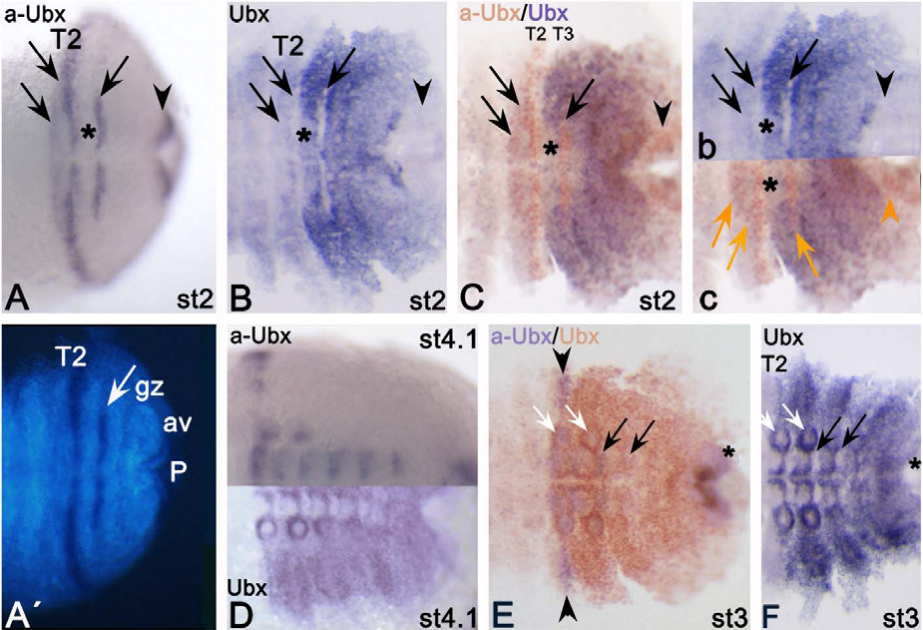
**Detection of complementary expression patterns of *Ubx *and *aUbx *in *Glomeris marginata***. **(A) **Stage 2 embryo stained for *aUbx*. Asterisk marks space free from transcripts. Arrows point to expression in (B); arrowhead points to expression in the anal valves. **(A') **DAPI counterstaining of the embryo shown in (A). **(B) **Stage 2 embryo stained for *Ubx*. Asterisk, arrows and arrowhead are in similar positions to (A). **(C) **Double staining with *aUbx *(orange) and *Ubx *(purple). Asterisk, arrows and arrowhead in similar positions to (A,B). **(B',C') **Cut out of embryos shown in (B,C) next to each other for ease of comparison of expression pattern. Asterisks, arrows and arrowheads are in similar positions (orange symbols in (C') point to expression of *aUbx*). **(D) **Stage 4.1 embryos expressing *aUbx *(upper) and *Ubx *(lower) respectively. **(E) **Posterior part of stage 3 embryo double-stained for *aUbx *(purple) and *Ubx *(orange). Arrowheads mark expression of *aUbx *in dorsal tissue. White arrows point to expression of *aUbx *in the tips of the legs. Note that *aUbx *is only at their base. Black arrows indicate segmental expression of *aUbx*. Asterisk indicates anal valves. **(F) **Embryo of same stage as in (E) expressing *Ubx*, with arrows and asterisk in similar positions.

The *Gm-aUbx *transcript is regulated in a similar, but complementary, specific pattern to that of *Gm-Ubx *(Figure 2, Figure 3, Figure 4). Expression of *aUbx *starts earlier (stage 0) than expression of *Ubx *(stage 0.2 or 0.3), but in a comparable posterior area. Double *in situ *hybridization to detect possible overlap of early *Ubx *and *aUbx *expression is not possible because the signal of *Ubx *is too weak in the early stages (Figure 2G-J). At stage 1, *Ubx *expression is still restricted to the posterior growth zone and is not present in the nascent segment T3 (Figure 2J), unlike the previously reported expression in T3 in stage 1.2 embryos [19]. At this stage, the anterior margin of *aUbx *is clearly more anterior (T2) than that of *Ubx *(T3). At stage 2, it becomes obvious that the expression patterns of *Ubx *and *aUbx *are indeed broadly complementary (Figure 4A-C). The stripe of *aUbx *expression extending into the dorsal tissue lies in the posterior of T2, and is thus anteriorly abutting the expression of *Ubx *(Figure 4A-C). The ventral *aUbx *stripe in T3 coincides with a lack of *Ubx *expression in this area (Figure 4A-D). Very faint expression of *Ubx *extends minimally into T2 ventrally (Figure 4B), and *aUbx *is weakly expressed anterior to this (Figure 4A-C). Whereas the ventral expression of *Ubx *at stage 4-6 becomes more complex, the expression of *aUbx *remains as stripes (Figure 4D), which are complementary to the expression of *Ubx*. (Figure 4D). The anterior shift of *aUbx *into the posterior of T1 (Figure 3A,B) coincides with a shift of *Ubx *expression into the complete ventral part of T2 (Figure 4F) [19]. The anterior border of dorsal *Ubx *is shifted towards the posterior compared with its anterior border in ventral tissue (Figure 4F) [19]. In dorsal tissue, the expression of *aUbx *still abuts the expression of *Ubx *and is hence also shifted towards posterior (Figure 3C, Figure 4E; also seen in Figure 4F for a stage 4 embryo).

### Transcript and expression of *Lithobius Ubx *and *aUbx*

**Figure 5:**
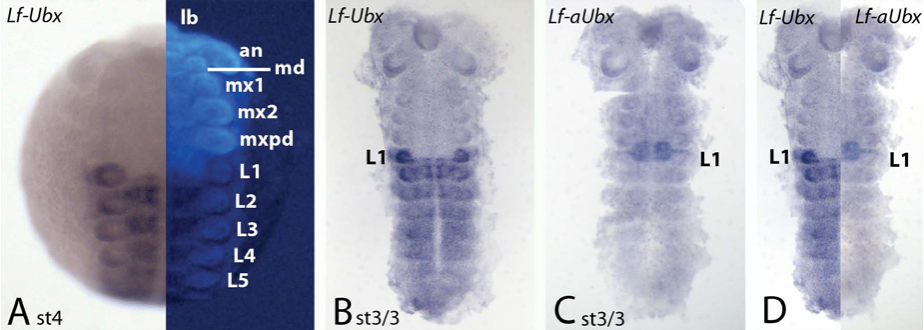
**Expression of *Lithobius Ubx *and *aUbx***. All embryos with anterior up. **(A) **Stage 4 embryo stained for *Ubx*. Left half is bright-field photograph; right half shows DAPI counterstaining. **(B) **Stage 3/3 flat-mounted embryo stained for *Ubx*. **(C) **Stage 3/3 flat-mounted embryo stained for *aUbx*. **(D) **Comparison of *Ubx *(left half) and *aUbx *(right half). Same embryos as in (b,c). an, Antenna; L1-L5, walking legs one to five; lb, labrum; md, mandible; mx1/2, first and second maxilla; mxpd, maxillipede.

The isolated fragment of *L. forficatus *(Uppsala/Sweden) *Ubx *is 93% (206 of 221 bp) identical with the orthologous sequence of *L. atkinsoni *[15] and 98% (64 of 65 bp) identical with the sequence of *L. forficatus *(UK) [22]. The expression pattern of *Lf-Ubx *is identical to that described for *L. atkinsoni *[15]). As expected from the data for *Glomeris *and *Strigamia*, the antisense transcript (*Lf-aUbx*) is also transcribed. The expression pattern of *Lf-aUbx *is complementary to that of *Ubx *and very similar to that of *Strigamia *antisense *Ubx *in embryos with 30 leg-bearing segments (LBS) (Figure 5) [9]. A broad central domain in the first walking leg segment (L1) abuts the anterior-most expression of *Ubx *which extends into the very posterior of L1. Dorsal to that, in the region of the developing legs, *aUbx *is expressed as a thin stripe at the border of the maxillipedal segment (mxpd) and L1 (Figure 5). We expect that the expression pattern of *Lf-aUbx *is more complex in older developmental stages [9].

### Detection of *aUbx *in arthropods other than myriapods

We investigated the possible expression of *aUbx *in members of other arthropod classes and an onychophoran. Sense probes of the same length as the antisense probes used for the detection of *Ubx *in *Tribolium *(Insecta), the two known *Ubx *paralogs in *Cupiennius *(Chelicerata) [16], and *Ubx *in *Euperipatoides *(Onychophora) failed to detect any transcripts. In all cases, positive controls detecting the *Ubx *signal were successfully probed with antisense probes in parallel experiments (data not shown).

## Discussion

### Conserved transcription and complementary expression of *Ubx *and *aUbx *supports myriapod monophyly

Sequence and expression data of *Ultrabithorax *are presently known from four myriapod species: the geophilomorph *Strigamia maritima *(Chilopoda) [9]; the lithobiomorph species *L. atkinsoni *and *L. forficatus *([15] and this study); and the pill millipede *G. marginata *(Progoneata) [19]. In all cases, the antisense DNA strand complementary to *Ubx *is transcribed and the expression pattern of the antisense transcripts (*aUbx*) is complementary to that of the sense transcript (coding transcript; *Ubx*) ([9] and this study). This finding suggests that complementary expression of sense and antisense transcripts generated from the *Ubx *locus is conserved between all myriapods.

Because *aUbx *expression has not yet been detected outside the Myriapoda, but has been detected in Chilopoda and Progoneata, it probably represents a synapomorphy for the Myriapoda, although this conclusion is dependent on the phylogenetic position of symphylans and pauropods [23-25]. This finding further supports myriapod monophyly, which is to date mainly based on nucleotide sequence data ([26,27] morphological data are still controversial in this context [10,25,28,29].

### Similarities of *Ubx *regulation in *Drosophila *and myriapods: evidence for a conserved mechanism?

The fact that *Ubx *and *aUbx *are expressed in conserved and complex complementary patterns strongly suggests that one (or its transcription) is involved in the regulation of the other. Striking similarities to the situation in myriapods can be found in *Drosophila*, in which transcription of *bxd *non-coding RNAs (ncRNAs) upstream of *Ubx *prevents transcription of the latter. This repression is probably caused by transcriptional interference as the *bxd *transcript(s) elongate into the region of *Ubx *promoters and prevent the binding of the transcription machinery [4,30]. As a result, *bxd *ncRNAs are expressed in a complementary pattern to that of *Ubx*, causing a mosaic-type expression pattern of *Ubx *within its overall expression domain [4,6]

A similar situation is found in myriapods, in which a putative ncRNA, *aUbx*, is expressed in a complementary pattern to that of *Ubx*. Like the *bxd *ncRNAs in *Drosophila*, *aUbx *also precedes expression of *Ubx*, and also as in *Drosophila*, expression of *Ubx *in myriapods occurs in the anterior of each segment and expression of *bxd *and *aUbx *occur in the posterior of each segment (this study, [9,31]).

The most obvious difference between the expression of *bxd *ncRNAs in *Drosophila *and *aUbx *in myriapods is that *aUbx *(or its promoter) is located on the complementary DNA strand in myriapods and not oriented in a tandem position to *Ubx *on the same strand. How can this disparity be explained if we assume that *aUbx *expression in myriapods is homologous to *bxd *expression in *Drosophila*?

**Figure 6:**
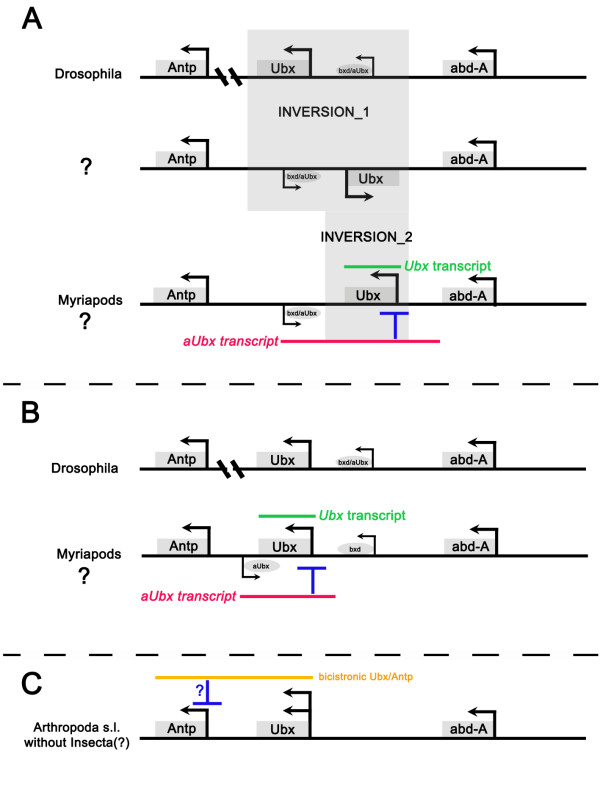
**Hypothetical interference of *aUbx *and *Ubx/Antp *transcription with *Ubx *and *Antp *expression**. **(A) **Inversion model explaining complementary expression patterns of *Ubx *and *aUbx *based on the hypothetical conservation of *bxd/aUbx *promoters. In a hypothetical ancestor of myriapods, *bxd/aUbx *and *Ubx *must be inverted (inversion_1) (indicated by question mark). A second inversion must have transferred *Ubx *back onto the leading strand. **(B) ***De novo *evolution of *bxd*-like *aUbx *promoter on the complementary strand upstream of Ubx. **(C) **Inferred negative interference of bicistronic transcription on *Antp *expression. If *Ubx/Antp *does not code for UBX protein, its expression may also repress monocistronic (translated) *Ubx *in *Glomeris*. Promoters and direction of transcription are indicated by arrows; genes are represented by shaded bars; crossbars indicate for the split Hox cluster in *Drosophila*; relevant transcripts are highlighted by coloured bars. Blue T-bars indicate suggested repressor function. Question marks indicate hypothetical functions/facts. Light shading indicates areas involved in inversion events.

The simplest explanation of this pattern would be to postulate an inversion event in the *Ubx *locus back in the stem lineage leading to the myriapods, placing the *aUbx (bxd) *promoter on the complementary strand (Figure 6A). Subsequent transcription through the promoter site(s) of *Ubx *in myriapods would then cause expression of *aUbx *in a complementary pattern. However, this would require a stage at which *Antp *and *Ubx *were on different strands, and as we show in this paper, bicistronic transcripts of *Ubx/Antp *and their splice versions (variants I and II) are conserved and thus are most probably of strong developmental importance, thus they are unlikely to have been separated in this way. A single inversion event putting *Ubx *alone on the complementary strand can also be excluded because of the presence of *Ubx/Antp *bicistronic transcripts that are very unlikely to be a result of a *trans*-splicing event (discussed below) [9,32]. An alternative to these unlikely possibilities is hat a new *bxd/aUbx *promoter site evolved on the complementary strand located between *Antp *and *Ubx *(Figure 6B). Functional studies or a fully sequenced genome, which could possibly help shed light on the role of *aUbx *transcription in myriapods and answer the question of whether the mechanisms suggested for *Ubx *regulation in myriapods are related to those in *Drosophila*, are currently not available.

### Alternative functions of *aUbx *expression

A number of theories have been suggested over the past few years to explain how noncoding antisense transcripts or bidirectional transcription may regulate the expression of the coding unit ([33] and references therein). A case of possible transcriptional interference displaying much similarity between *Drosophila *and myriapods has been discussed in the previous section. However, although this possibility appears to be likely, *aUbx *or its transcription could nevertheless also act differently. We therefore summarize and discuss some of those mechanisms in the light of our data.

First, transcription of the antisense strand can cause epigenetic modifications, methylation of sense-strand promoters, and conversion of the chromosome structure, causing repression of gene transcription on the sense strand [34]. Epigenetic modification could explain or cause the complementary pattern of *Ubx *and *aUbx *if *aUbx *represses the transcription of *Ubx *in tissues or cells that are generally *Ubx*-competent.

Second, transcriptional interference can also occur via promoter collision, when RNA polymerases meet on opposite strands and cannot pass each other. This can cause the premature termination of one or both transcripts [30,35].

Third, sense and antisense transcripts could form double-stranded (ds)RNA, a source for small interfering RNAs that would mediate RNA interference (RNAi) [36]. The complementary expression pattern of *Ubx *and *aUbx *would be explainable by the rapid degeneration of *Ubx *due to perfectly matching miRNAs descendent from the possible *Ubx*-*aUbx *dsRNA [37].

The fact that *aUbx *is expressed significantly earlier than *Ubx *may also have important implications on the regulatory mechanisms discussed. It would guarantee the immediate binding of incorrectly expressed *Ubx *to pre-existing *aUbx *in an RNAi-based mechanism, or provide a head start for transcription of *aUbx *in cases of transcriptional interference. In the case of epigenetic modification, it would prevent the later transcription of *Ubx *by silencing its promoter(s).

### A 21 bp repeat in the *Ultrabithorax *3' UTR of *Glomeris*

We discovered a repetitive sequence of exactly 21 bp (Figure 1D) in the 3' UTR of *Ubx*. This sequence most probably represents a minisatellite (or short sequence repeat; SSR) common in bacterial and metazoan genomes [38]. It may represent multiple recognition sites for micro (mi)RNAs [39]. Alternatively, it could represent an ORF encoding a small 113 amino acid protein, possibly involved in the regulation of *Ubx*. The finding of an SSR could generally also be of interest for investigating population genetics in *Glomeris *[40].

### Presence of *Ubx/Antp *bicistronic transcripts in myriapods, crustaceans and onychophorans, but not in insects?

The finding that bicistronic transcripts of *Ubx *and *Antp *(*Ubx/Antp*) are present in myriapods and crustaceans suggests that this represents a conserved state of at least the Mandibulata or potentially the Arthropoda. Despite this, we failed to isolate *Ubx/Antp *fusion transcripts from the beetle *T. castaneum*. The latter may merely represent a loss in higher insects that finally allowed the Hox complex to split between *Ubx *and *Antp*, as is the case in *Drosophila melanogaster *[41]; however, in *Tribolium*, the Hox cluster is still intact [8]. Alternatively, it may represent the early loss of *Ubx/Antp *in the stem lineage of the insects or hexapods. If the hexapods have evolved from a crustacean ancestor (as in the Pancrustacea theory), a loss of *Ubx/Antp *may be present in the suggested recent sister-group crustacean orders Remipedia and/or Cephalocarida [42]. The presence of *Ubx/Antp *fusion transcripts in an onychophoran shows that the evolutionary origin of bicistronic transcription of *Ubx *and *Antp *dates back to the common ancestor of onychophorans and euarthropods, suggesting that *Ubx/Antp *is also likely to occur in chelicerates.

Interestingly, only the short splice variant II (Figure 1B,B') has been isolated from myriapods. We therefore believe that variant I may be lacking in myriapods exclusively, again supporting myriapod monophyly. However, we are aware that negative results are less reliable arguments than positive results, and therefore we can only see the lack of splice variant I in myriapods as minor evidence for monophyletic Myriapoda.

The presence of the *Ubx/Antp *splice variant II in onychophorans, crustaceans and myriapods argues against a mere genomic rearrangement in a population of *Ubx *as suggested for the centipede *Strigamia *[9], but rather suggests an important and conserved role in Hox gene regulation across the Arthropoda.

### Conserved regulatory aspects of *Ubx/Antp *expression

In crustaceans, bicistronic transcripts of *Ubx/Antp *are not (*Daphnia*) or only partially (only *Ubx *in *Artemia*) translated. Expression of the translated monocistronic transcripts, and therefore the protein, differs significantly from expression of *Ubx/Antp *[11]. It is tempting to speculate that transcription of *Ubx/Antp *under control of the *Ubx *promoter interferes with the proper transcription of monocistronic *Antp *in these crustaceans.

The conserved appearance of *Ubx/Antp *in arthropods and onychophorans suggests their involvement in the regulation of *Ubx*, *Antp *or both *Hox *genes. In particular, repression of *Antp *via *Ubx/Antp *transcription appears likely, not least because the transcript is apparently spliced in such a way that it lacks most of its coding capacity (variant II).

For *Glomeris *and *Euperipatoides*, it is unclear whether the detected expression patterns of *Ubx *and *Antp *are a result of mono-or bicistronic transcription. However, in both, as in crustaceans [11], the *Ubx/Antp *transcript is probably under control of the *Ubx *promoter, as the expression pattern of *Ubx/Antp *is identical with that of *Ubx *(not shown). Thus, it is possible *Ubx/Antp *contributes to or even replaces monocistronic *Ubx *expression in *Glomeris *and *Euperipatoides *as it does in *Artemia *[11]. If part of the detected mRNA expression patterns of *Ubx *and *Antp *[19] is a result of *Ubx/Antp*, it might not correlate with the protein pattern. Specific antibodies to detect UBX and ANTP protein are not available, and the crossreacting antibody FP6.87 [43] does not detect UBX in *Glomeris *(data not shown). Further investigation is thus needed to unravel the role of *Ubx/Antp *transcription in arthropods.

### Regulation of limb development in *Glomeris*

*Ubx *expression is likely to be involved in the delayed outgrowth of the walking legs posterior to T3 in *Glomeris *by repressing *Distal-less *(*Dll*) as shown for other arthropods [44-46]. The finding that *aUbx*, a possible repressor of *Ubx *(as discussed above), is strongly expressed in the tips of the legs in T2 and T3 further supports this view, suggesting that the absence of *Ubx *is indeed crucial for the accelerated development of walking legs in T1 to T3 in *Glomeris *[19] . The exclusion of *Ubx *from the distal part of the legs possibly caused or supported by *aUbx *could represent a developmental novelty in the 'battle' of appendage growth in *Ubx*-expressing segments. In *Strigamia *and *Lithobius*, *Ubx *seems not to repress *Dll*, possibly because of a number of phosphorylation sites in the C-terminal end of the protein that interfere with the assumed repressor function of Ubx on Dll [19,45]. Consequently, there is no need to keep the tips of the legs free from *Ubx *or, in other words, to express *aUbx*.

## Conclusions

A number of conserved aspects of *Ubx *and *Antp *regulation are found across the Arthropoda. Repression of *Ubx *transcription, and thus formation of a complex segmental pattern of *Ubx *expression, may depend on transcriptional interference as shown for *Drosophila*, and suggested and visualized by *aUbx *expression in *Glomeris*. Furthermore, bicistronic transcription of *Ubx *and *Antp *and subsequent splicing of these transcripts as shown for Crustacea, Myriapoda and Onychophora, but possibly not Insecta, suggests that *Ubx/Antp *transcription is an important ancestral feature of Hox gene regulation as well. As shown for Crustacea, runthrough transcription and subsequent nontranslation of *Ubx/Antp *may compete with the proper transcription of the (translated) monocistronic *Ubx *and *Antp *transcripts [11], and thus transcriptional interference via *Ubx/Antp *transcription might contribute to a defined protein expression pattern within areas of ubiquitously expressed Hox gene mRNA. Presence of *aUbx *transcription and the possible lack of *Ubx/Antp *splice variant I in myriapods represent possible synapomorphies for the Myriapoda.

## Competing interests

The authors declare that they have no competing interests.

## Authors' contributions

RJ carried out the experiments, wrote the first draft of the manuscript and was mainly responsible for the experimental outline. GEB was involved in drafting the final version of the manuscript and discussed the experimental outline. GEB also initiated work on *Euperipatoides*.
